# On-Site Measurement of Fat and Protein Contents in Milk Using Mobile NMR Technology

**DOI:** 10.3390/molecules27030583

**Published:** 2022-01-18

**Authors:** Morten K. Sørensen, Nicholas M. Balsgart, Michael Beyer, Ole N. Jensen, Niels Chr. Nielsen

**Affiliations:** 1 Nanonord A/S, Skjernvej 4A, DK-9220 Aalborg, Denmark; balsgart@gmail.com (N.M.B.); mbe@nanonord.dk (M.B.); oj@nanonord.dk (O.N.J.); 2 Department of Biological and Chemical Engineering, Aarhus University, Finlandsgade 12, DK-8200 Aarhus, Denmark; 3 Interdisciplinary Nanoscience Center (iNANO) and Department of Chemistry, Aarhus University, Gustav Wieds Vej 14, DK-8000 Aarhus, Denmark

**Keywords:** milk, fat, protein, magnetic resonance, low-field NMR, benchtop NMR, contrast agent, quantification, on-farm analysis

## Abstract

Robust and easy-to-use NMR sensor technology is proposed for accurate, on-site determination of fat and protein contents in milk. The two parameters are determined using fast consecutive ^1^H and ^35^Cl low-field NMR experiments on milk samples upon the 1:1 addition of a low-cost contrast solution. Reliable and accurate measurements are obtained without tedious calibrations and the need for extensive database information and may readily be conducted by non-experts in production site environments. This enables on-site application at farms or dairies, or use in laboratories harvesting significant reductions in costs and time per analysis as compared to wet-chemistry analysis. The performance is demonstrated for calibration samples, various supermarket milk products, and raw milk samples, of which some were analyzed directly in the milking room. To illustrate the wide application range, the supermarket milk products included both conventionally/organically produced, lactose-free milk, cow’s, sheep’s and goat’s milk, homogenized and unhomogenized milk, and a broad nutrient range (0.1–9% fat, 1–6% protein). Excellent agreement between NMR measurements and reference values, without corrections or changes in calibration for various products and during extensive periods of experiment conduction (4 months) demonstrates the robustness of the procedure and instrumentation. For the raw milk samples, correlations between NMR and IR, NMR and wet-chemistry, as well as IR and wet-chemistry results, show that NMR, in terms of accuracy, compares favorably with the other methods.

## 1. Introduction

Milk is a world-wide major food resource and as such is subject to extensive analysis requirements to ensure quality, nutritional yield, animal health, and production output. In modern farming, routine analysis is performed on milk delivered to dairies, and regular analyses are performed on the milk from individual animals providing indications on the nutritional balance of the milk as well as the health of the animal. For farmers, the milk analyses also provide essential information to control and optimize feeding and breeding strategies. At dairies and for milk manufacturers, milk analysis is performed as an essential part of the process control. Milk analysis is also important for product development, product price, quality assurance and research. The main parameters in milk analysis are typically the contents of fat and protein. Often the fat and protein contents are used to calculate the milk’s value directly determining payment to farmers for delivered milk.

Today, the vast majority of the huge number of milk analyses are performed using infrared (IR) spectroscopy with the instrumentation calibrated to database information by wet-chemistry (WC) methods like gravimetric analysis for fat content and Kjeldahl or Dumas analysis for protein content [1,2,3]. IR analysis provides fast determination (in the order of 30 s) of several parameters using benchtop instruments or on-line instruments for monitoring at production facilities. However, it is well known that IR analyses may suffer from calibration drifts and poor accuracy when not subjected to frequent and careful calibration to correctly chosen calibration samples of a similar type, origin, and nutritional value as the samples subject to analysis. Accordingly, IR instruments for measurements of milk samples are typically only operated by expert users from commercial laboratories, dairies, or research facilities.

Wet-chemistry analysis either performed directly for milk sample analysis or forming the calibration basis for IR analysis are generally considered costly, time-consuming and demanding on required procedures and chemicals. It is worth noting that also the wet-chemistry methods are associated with assumptions, in particularly for protein analysis which relies on the total nitrogen content scaling to protein contents by a fixed number [3,4], and are associated with analysis uncertainty as all measuring methods. It also generally applies that any method is not better than the provided sample and representativity of the typically very small subsamples taken for actual analysis.

In this work, we introduce mobile, low-field nuclear magnetic resonance (NMR) sensor technology for robust, accurate, and operationally simple on-farm or laboratory quantification of fat and protein contents in milk performed directly on the milk samples upon the addition of a simple contrast solution. Since NMR relies on the direct detection of the response of isotopes (upon radio-frequency perturbation in a strong external magnetic field), NMR has the great advantage relative to optical methods (such as IR) in offering quantitative results for the bulk volume of the analyzed material. This implies that NMR has no requirement for an optical window or consideration of penetration depths, and NMR does not require the establishment and maintenance of extensive databases suitable for specific types of samples as applies to IR.

Low-field and mobile NMR is currently undergoing tremendous development for laboratory analysis [5], and is now gradually also finding applications for production site analysis, which demand more robust and easier-to-apply instrumentation. We have, over the past years, developed multinuclear, mobile NMR sensors for production-line and laboratory analysis in as wide application areas as the quantification of nutrients (nitrogen, phosphorus, potassium) in animal slurry [6,7] and waste water streams, salts (sodium and chloride) in food products, protein and phosphorus in livestock feed [8], onboard monitoring of catalytic fines (aluminum) in heavy fuel oil in the shipping industry [9], oxygen measurements [10], and benchtop solid-state NMR [11]. This instrumentation allows high sensitivity and fast switching between all relevant NMR isotopes, non-expert-user operations, and robustness for on-site applications.

Aimed at accurate, on-site quantification of fat and protein contents in milk, we propose here an easy protocol allowing direct quantification using low-field NMR on milk samples upon the 1:1 addition of a simple contrast solution and temperature stabilization. The contrast solution is composed of a 6% regular salt (NaCl) solution containing 2% sodium ferric ethylenediaminetetraacetate (Fe-EDTA), which is easily available at a low cost and is safe to use for non-experts. Relaxation agents (like Fe-EDTA) are well-known and widely applied for contrast improvements in magnetic resonance imaging [12,13] and for sensitivity improvements in NMR spectroscopy through the enhancement of the relaxation rate of interacting molecules, thereby enabling the conduction of faster experiments [14,15].

In the context of milk, the presence of paramagnetic iron (Fe(III)) significantly increases the relaxation rate of protons in water, while relaxation of protons in fat remains largely invariant due to a much lower degree of interaction with the iron ions. This easily-controlled editing of relaxation rates isolates the slow-relaxing fat components in the signal acquired using a standard Carr–Purcell–Meiboom–Gill (CPMG) type [16,17] echo-train of pulses, which enables stable and precise quantification of the fat signal with minimal interference from the water proton signal. Under normal circumstances, without contrast solutions, the water dramatically dominates the signal preventing precise information about the fat content unless the water is removed by drying prior to measurement [18,19]—which, however, tend to be impractical and time-consuming as it requires all water to be evaporated or the part left quantified—or potentially using instrumentation equipped with very strong field gradients [19,20,21] which needs careful calibration to discriminate components in terms of spin diffusion.

The proposed ^35^Cl NMR method for quantification of protein relies on ^35^Cl transverse relaxation rate (*R*_2_ = 1/*T*_2_; *T*_2_ is the transverse relaxation time), which we, for milk samples, have found to correlate well with the actual content of protein with minimal interference from varying amounts of other constituents such as fat and lactose. We hypothesize that the underlying mechanism is the electrostatic interaction between chloride ions and the partly charged sites on the protein. This interaction with large particles introduces a linear (at least in the desired concentration range) change of the relaxation rates for all dissolved chloride ions due to the fast chemical exchange of interacting and free chloride ions. The addition of NaCl in relatively high concentrations facilitates fast detection of the otherwise relatively insensitive chlorine isotope, and furthermore ensures a sufficient amount of free chloride ions relative to the numbers of protein interaction sites.

Combining the easy sample handling procedures and the detection principles outlined above with robust and accurate multinuclear NMR sensors provides a new highly reliable method for the quantification of fat and protein in milk, with easy operation and without the demands for frequent calibrations nor reference to large databases. The NMR measurements can be conducted by non-experts in a rough production-line environment providing immediate and accurate results on the site of application (e.g., directly at farms, at dairies, or in laboratories). It can be applied in all situations, including situations where demanding calibration and maintenance of alternative IR instrumentation cannot be fulfilled. In a different approach, the proposed method may serve as an alternative to wet-chemistry methods, possibly stepping in as a reference method for the calibration of on-line IR instruments. We also envisage that the proposed method may be extended to applications in a flow-system with the automatic injection of contrast solution and temperature stabilization. This, however, is beyond the scope of this paper.

To demonstrate the performance of the low-field NMR relative to IR and laboratory methods, we have evaluated various supermarket milk products and raw milk samples (some analyzed on-farm in the milking room). The samples were collected and evaluated over a period of about 4 months without corrections or changes in calibration, which along with the broad range of milk types serves to illustrate the broad range, stability, and robustness of the technology.

## 2. Results and Discussions

### 2.1. Calibration Samples

To obtain a reliable calibration, using samples with the best-possible known true fat and protein contents, a set of milk calibration samples (commercially available for calibration of IR instruments) were purchased from Eurofins Milk Testing (Vejen, Denmark). The calibration samples were three different pasteurized and homogenized samples and two different raw milk samples. We note that the calibration samples from the provider were added Bronopol preservative and a dye, giving the samples a green color which we, however, do not ascribe any significant perturbation on the measurements due to the low concentration. For better coverage of the fat and protein concentration range of interest, we also analyzed five dilutions of the calibration samples mixed 1:1 with demineralized water.

**Figure 1:**
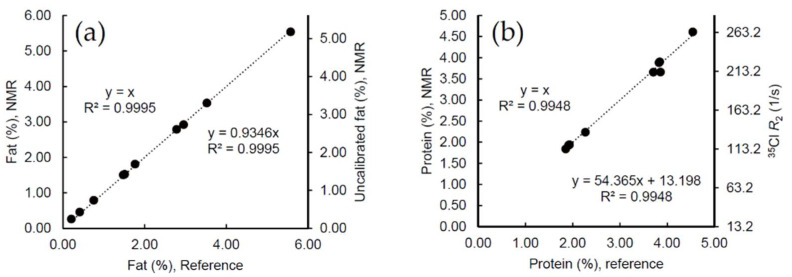
Calibration curves for low-field NMR fat and protein analysis using commercial calibration samples designed for calibration of IR instruments. (**a**) NMR fat results correlated to fat reference values with calibrated NMR fat results on the primary axis and the uncalibrated NMR fat measurement obtained directly by ^1^H NMR intensities (calculated as the fat-H to total-H ratio times the applied dilution factor of 2 times 100%) on the secondary axis. (**b**) Calibrated NMR protein results (primary axis) and ^35^Cl NMR relaxation rates (*R*_2_, secondary axis) correlated to protein reference values. See text for description of samples. The correlation equations to the left and right correspond to the left and right vertical axis, respectively.

Figure 1 illustrates NMR measurements of fat and protein for the calibration samples correlated to the reference values provided by Eurofins. The primary vertical axis shows the calibrated direct correlation with reference values, while the secondary axis relates 1:1 to the observed NMR parameter. For fat, the latter is the ^1^H NMR intensity measured for the protons in fat divided by the total ^1^H NMR total signal intensity and scaled with the dilution factor (1:1 mix of milk with contrast solution) times 100 percent, and the given equation (to the right) correlates this ratio to the reference values. Similarly for protein, the secondary axis gives the measured ^35^Cl relaxation rate, which, through the equation to the right in the figure, is correlated to the reference values.

The correlations in Figure 1 serve to calibrate the NMR results for fat and protein. We note that, for fat, the correlation for the directly measured ^1^H signal is almost equal to the reference values, which means that only a minor scaling factor is required to adjust uncalibrated signals to reference values. The scaling factor close to 1 is advantageous since it leaves possible effects from unexpected instrumentational perturbations on the calibration to an absolute minimum (thus providing a very stable calibration).

### 2.2. Supermarket Milk Products

As a simple means to illustrate the expected broad performance and robustness over various product types, origin (animal), seasonal variation, and wide range of fat and protein contents, a selection of milk products were purchased from local supermarkets. The products include regular cow’s milk with various fat contents from skimmed milk (0.1% fat) to whole milk and coffee cream (9% fat), goat’s and sheep’s milk, homogenized and unhomogenized milk, fresh and long-life (UHT-treated) milk, organically and conventionally produced milk, and also lactose-free milk, milk products for infants, and milk from cows only fed with grass (see Table S1 in the Supplementary Material for a full list of samples).

**Figure 2:**
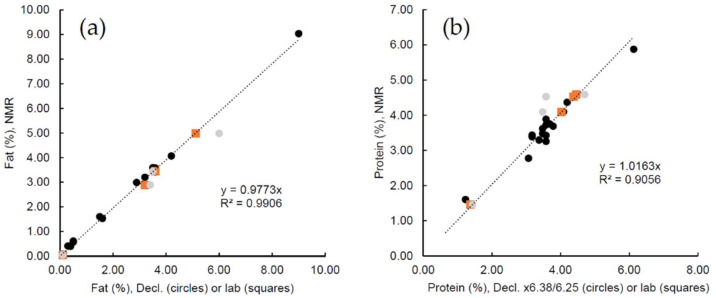
(**a**) Fat and (**b**) protein content (in %) obtained using low-field NMR (vertical axis) compared to declared (circles, 20 samples) and laboratory (squares, 4 samples) content (horizontal axis) for various supermarket milk products (see Table S1 for a list of included products and content). Grey circles indicate the declared contents for the 4 samples with laboratory results. For the shown trendlines, the laboratory results were used for the four samples where existing.

Figure 2 shows the NMR results for fat and protein correlated to declared contents (circles, all samples) and laboratory analyses (squares, four samples) for the supermarket milk products. Generally, the NMR results are in excellent agreement with the expected contents of the various products (laboratory results when existing). We note that declared contents are average values and actual contents in the specific packages may be subject to variations including differences in individual aliquots and seasonal variation of the raw milk. This obviously may contribute to the deviation between NMR results and declared contents. To address this issue, we had an additional laboratory analysis performed for four samples showing deviations between NMR results and declared contents with a difference larger than 0.4% for either fat or protein (grey circles in Figure 2). Aliquots of these samples were sent to wet-chemistry analysis at an external laboratory (AGROLAB LUFA GmbH, Kiel, Germany), where they were analyzed using the Röse–Gottlieb method for fat and the Kjeldahl (N × 6.38) method for protein. The laboratory results (squares in Figure 2) support the NMR results and may confirm that, for some products, the declared contents were not accurately representing the actual content in the milk product.

With these remarks in mind, the quality of the correlations in Figure 2 for a variety of supermarket samples are confirmative of the wide application range of the NMR measurements. We should emphasize that the NMR measurements were conducted without any correction or calibration changes between products or during the period of four months over which the shown measurements were conducted.

### 2.3. Raw Milk Samples

To demonstrate the performance for the analysis of raw milk, we examined 30 samples of untreated milk from cows at the Danish Cattle Research Centre, Aarhus University. The samples were taken from 30 dairy cows (Danish Holsteins) at a morning milking, along with samples taken for regular analysis for individual cows (IR analysis at Eurofins laboratory, Vejen, Denmark), and stored cold (2–5 °C) until preparation of NMR sample tubes. To illustrate the performance also in a rough environment, the measurements of the first 12 samples (first duplicate samples) were performed directly in the milking room (see Graphical Abstract image), which did not reveal differences in performance compared to other measurements. Individual IR laboratory results were provided for all 30 samples. Additionally, upon taking small subsamples used for NMR analysis, the remaining aliquots of 15 samples (unequal sample numbers) were sent to an external laboratory (AGROLAB LUFA GmbH, Kiel, Germany) for wet-chemistry (WC) analysis using the Röse–Gottlieb method for fat and the Kjeldahl (N × 6.38) method for protein.

**Figure 3:**
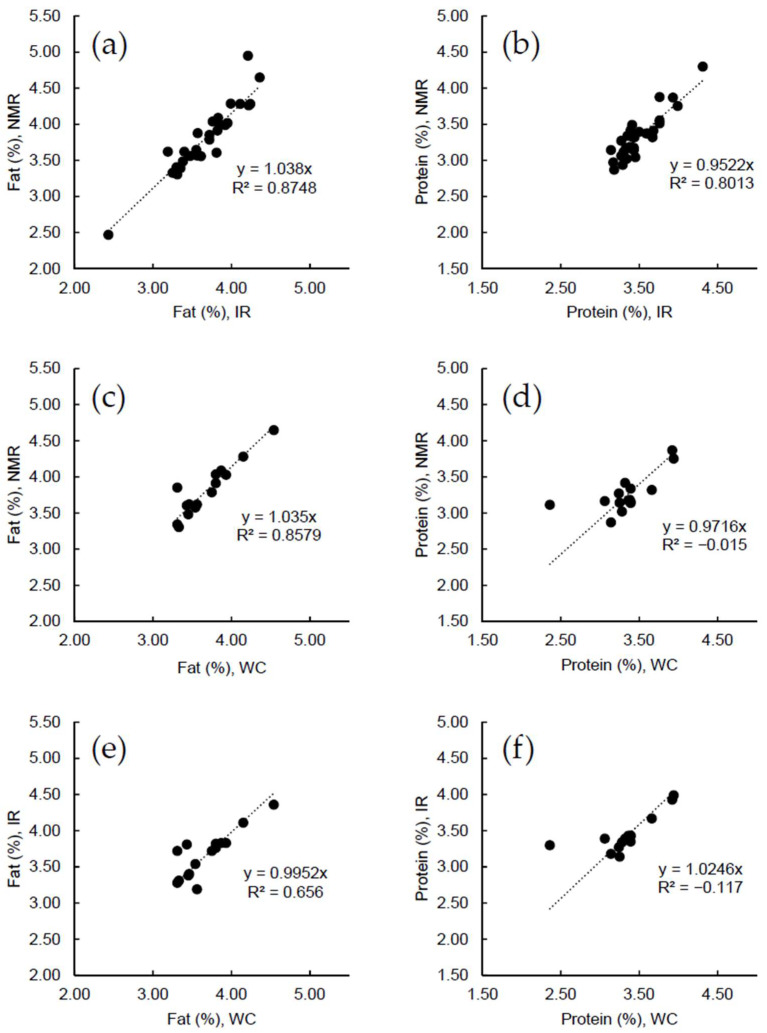
Correlation of NMR, IR, and wet-chemistry (WC) determined content (in %) of fat (**a**,**c**,**e**) and protein (**b**,**d**,**f**) content in raw milk samples. (**a**,**b**) NMR vs. IR results (30 samples). (**c**,**d**) NMR vs. WC results (15 samples). (**e**,**f**) IR vs. WC results (15 samples). See text for details on the NMR, IR and WC methods.

Figure 3 correlates results for fat and protein quantification obtained using NMR vs. IR (Figure 3a,b), NMR vs. WC (Figure 3c,d), and IR vs. WC (Figure 3e,f), respectively. In all cases, we obtain correlations with slope with variations within 5% relative to unity, but also include significant spread which relate to the measurement accuracy of the correlated analysis methods. For protein, the WC result of 2.36% for one sample show a large deviation from the better matching IR result of 3.30% and NMR result of 3.12%. This could indicate inaccuracy or a mistake in the result reported from the external laboratory.

Table 1 provides the standard deviations (STDs) for the differences in fat and protein content observed using different methods as calculated for NMR vs. IR, NMR vs. WC, and IR vs. WC results. For fat, the STDs are quite similar for all three pairs of methods, however, slightly better for those including NMR. For protein, NMR vs. IR are clearly better due to the outlier in the WC results. If the outlier is excluded, the STDs for protein are 0.14% for NMR vs. WC and 0.10% for IR vs. WC. The results provides evidence that the NMR analysis provides a similar accuracy to the IR and WC analysis for the analyzed samples, but a more comprehensive study using more samples is required to judge the exact performance of each method. However, we should note that the correlation between NMR results and reference data were significantly better for the calibration samples (Section 2.1), which we ascribe to more reliable reference values for the calibration samples since these are presumably subject to carefully analysis at the supplier. This also supports the indication that NMR may provide an equal or superior accuracy to IR and WC measurements.

We emphasize that all analytical approaches (including both NMR, IR, and WC results) are obviously associated with uncertainties covering both intrinsic uncertainties of the methods, deviations in the actually analyzed part of subsamples, and all possible technical issues. The STDs given in Table 1 cover contributions of all kinds from both methods involved for each value. Specific contributions from the respective methods and sampling may be examined further in a larger correlation study as for example recently performed for quantification of nutrients in animal slurry [7].

### 2.4. Precision

To evaluate the precision of the NMR measurements, all samples were analyzed for two duplicate samples and with two repeated measurements for each sample (see Material and Methods). Table 2 shows calculated STDs for the repeated measurements and for duplicate samples given for specified sample types and in groups of homogenized/unhomogenized milk. The STDs are calculated using $s_{dupl}=\sqrt{\frac{1}{2n}\sum_{i=1}^{n}{\left(x_{i}^{A}-x_{i}^{B}\right)}^{2}}$, with $s_{dupl}$ being the STD for duplicates or repeated measurements as given in reference [22], while $x_{i}^{A}$ and $x_{i}^{B}$ are the two results for the *n* duplicate/repeat pairs.

The results in Table 2 reveal that NMR provides an excellent precision for quantification of fat and protein in milk. For fat, on all samples, the STDs on repeated measurements (measuring time 1 min) are 0.02–0.04%, while, for homogenized samples, the STDs on duplicate samples are as low as 0.01–0.03%. We note that for each duplicate sample the measurement reflects the average of two repeated evaluations (2 min in total on each sample for fat). For fat on unhomogenized samples, the STDs for duplicates are higher (0.05–0.21%), which most likely occur due to inhomogeneity in the samples which may be particularly visible for the raw milk samples. For protein, on all samples, STDs on repeated measurements (3 min) are 0.09–0.19%, while on duplicate samples (measuring time 6 min) the STDs are 0.05–0.15%. For both fat and protein, the results show that the measuring times of 1 min per sample for fat and 3 min per sample for protein seems appropriate for most practical applications with the current setup and instrumentation. For low-fat products like skimmed milk, an improved precision on fat measurements can be obtained using a longer measuring time and/or an alternatively prepared sample aiming at higher Fe-EDTA concentration and less dilution. For example, using 5 min measurements on samples with 4% Fe-EDTA added as powder to skimmed milk, we obtained an STD of 0.005% for repeated fat measurements.

Especially for fat, the results also indicate that the reproducibility of measurements may benefit from sample homogenization prior to sampling as also expected for all other accurate methods. Alternatively, in all cases, the obtained accuracy obviously benefits from the evaluation of replicate samples, minimizing the uncertainty induced by sampling. For NMR, the use of replicate samples may allow the shortening of individual measuring times to a total duration aligning with the times given above, since the contribution from intrinsic noise of NMR measurements is also minimized across analysis of multiple samples.

## 3. Materials and Methods

### 3.1. NMR Instrument and Pulse Sequences

All NMR experiments were conducted using a mobile Tveskaeg NMR instrument (Nanonord A/S, Aalborg, Denmark). The instrument includes a 1.5 T permanent magnet constructed as a cylindrical Halbach array and a field-programmable gate array (FPGA) digital spectrometer in connection with an rf probe with an inner bore diameter of 9.2 mm. The instrument is tunable for the full frequency-range of NMR active isotopes and is designed for very fast tune-changes between relevant isotopes (e.g., ^1^H and ^35^Cl for the present application), and operates at a magnet temperature stabilized at 39.0 °C. All signal acquisitions are performed in-between pulses in a train of 180° refocusing pulses (observing the coherent echoes) implemented as ^1^H Carr–Purcell–Meiboom–Gill (CPMG) [16] and ^35^Cl quadrupolar CPMG (QCMPG) [17] pulse sequences for fat and protein measurements, respectively. The ^1^H measurements were acquired at 64.2 MHz using 1408 echoes (gathered in 128 groups of 11 summed echoes) with an echo spacing of 300 µs, a recycle delay of 2.5 s, and rf pulses of lengths 9 and 18 µs for π/2 and π pulses, respectively. The ^35^Cl experiments were acquired at 6.3 MHz using 90 echoes (gathered in 30 groups of 3 summed echoes, with two initial echoes unacquired), an echo spacing of 240 µs, a recycle delay of 300 ms, and rf pulses of lengths 22.5 and 45 µs for π/2 and π pulses, respectively.

### 3.2. Contrast Solution

Contrast solutions were prepared as 8 g Fe-EDTA (Sigma-Aldrich, St. Louis, MO, USA) and 24 g NaCl (VWR Chemicals) to 368 g demineralized water, with Fe-EDTA dissolved before adding NaCl.

### 3.3. Conduction of Measurements

Practically, each NMR measurement of fat and protein was conducted by the following steps: (*i*) Milk and contrast solution were mixed in a 1:1 ratio to a total of 5–12 mL from which about 2.1 mL (42 mm height in NMR tube) were transferred to a NMR tube of perfluoroalkoxy alkane (PFA) with inner diameter of 8 mm; (*ii*) The filled NMR tubes were temperature stabilized in a water bath at 39 °C for minimum 20 min; (*iii*) The tubes were inserted into the NMR instrument and measurements started to provide the final results.

To obtain statistical data, duplicate NMR tubes were prepared for every sample, and each duplicate sample were measured for two times 1 min ^1^H NMR (for fat) and two times 3 min ^35^Cl NMR (for protein). The shown NMR results are mean results of two repeated measurements of two duplicate samples (in total, four times 1 min for fat and four times 3 min for protein), except for the calculated STDs in Table 2, as discussed in Section 2.4.

### 3.4. Signal Processing

Signal processing was performed using simple Matlab [23] routines on the integrated echo-train signal intensities. For fat, two consecutive exponential fits were performed with the signal from non-fat (mainly water, but presumably also small molecules like lactose) and fat components, each approximated by an exponential decay. First, a bi-exponential fit (*y* = *I*_water_ exp(*−t*/*T*_2,water_) + *I*_fat_ exp(*−t*/*T*_2,fat_)) was performed on the echo-train data with the exclusion of the first 15 echo groups (165 echoes, roughly 50 ms). This provided the intensity of the fat signal (*T*_2,fat_ ~ 200–300 ms) with minimized interference from the water signal (*T*_2,water_ ~ 20 ms). Thereafter, a mono-exponential fit was performed on the first 15 echo groups (with the calculated fat signal decay subtracted) to obtain the non-fat part of the total ^1^H NMR signal. This allows the calculation of the ratio between the fat ^1^H NMR signal to the total ^1^H NMR signal, using the fat intensity from the first fit and the total intensity as the sum of the fat intensity and the non-fat intensity obtained from the second fit. While this is obviously also directly achievable from a bi-exponential fit on the full echo-train signal, we found the serial two-fits procedure to be more robust.

For protein, the chlorine relaxation rate (*R*_2_) is obtained by a mono-exponential fit (*y* = *A* exp(*−tR*_2_)) to the ^35^Cl NMR echo-train signal decay. In all cases, the performed exponential fits are standard least squares fits.

## 4. Conclusions

In this work, we have introduced novel measurements of fat and protein contents in milk using mobile, low-field NMR sensor technology suitable for on-site application and without tedious calibration requirements. The evaluation of a variety of milk types including calibration samples, a variety of supermarket milk products and raw milk revealed a broad and robust performance with excellent precision, and indications of an accuracy which is equal to or superior than current IR and WC methods. The intended main application is on-site analysis at farms or dairies, but may also include applications at laboratories for easy, fast and reliable analysis.

## 5. Patents

Patents applications have been filed for fat determination and for protein determination including the methods presented in this work.

## Figures and Tables

**Table 1:** Standard deviations (STDs; in %) on the difference between results of two methods calculated for NMR vs. IR, NMR vs. WC, and IR vs. WC results for the raw milk samples. For NMR vs IR, the STDs are given for the 15 samples also covered by laboratory results in order to have best comparison (however, we note that values obtained with all 30 samples included are very similar to those with 15 samples included).

	NMR vs. IR	NMR vs. WC	IR vs. WC
Fat (%)	0.15	0.14	0.19
Protein (%)	0.13	0.27	0.25

**Table 2:** STDs for repeated measurements and measurements of duplicate samples by NMR for fat and protein, respectively. See text for description of calculation.

	Fat (%)		Protein (%)	
	Repeats	Duplicates	Repeats	Duplicates
Homogenized calibration samples	0.02	0.01	0.12	0.07
Unhomogenized calibration samples	0.03	0.05	0.09	0.05
Homogenized supermarket milk	0.04	0.03	0.10	0.07
Unhomogenized supermarket milk	0.03	0.08	0.19	0.15
Raw milk (unhomogenized)	0.02	0.21	0.09	0.11

## Data Availability

The data presented in this study are available on request from the corresponding author.

## Supplementary Materials

The following supporting information can be downloaded. Table S1: List of supermarket milk products with declared contents, NMR results (mean of duplicate samples) and laboratory results for fat and protein, respectively. For protein, the declared contents are given as the package declaration scaled by k = 6.38/6.25, since package declarations are based on an assumed Jones factor of 6.25.
