# Supplementary material for: On-Site Measurement of Fat and Protein Contents in Milk Using Mobile NMR Technology
Source: Molecules. 2022 Jan 18;27(3):583. doi: 10.3390/molecules27030583 (PMC8839330; doi:10.3390/molecules27030583)
Supplement: Supplementary file 1 [file molecules-27-00583-s001.zip › molecules-1537317-supplementary.pdf]

Supplementary Material for:

On-site Measurement of Fat and Protein Contents in Milk using Mobile NMR Technology

Morten K. Sørensen [1,2,3,*], Nicholas M. Balsgart [1], Michael Beyer [1], Ole N. Jensen [1] and Niels Chr. Nielsen [1,*,†]

[1] Nanonord A/S, Skjernvej 4A, DK-9220 Aalborg, Denmark; balsgart@gmail.com (N.M.B.); mbe@nanonord.dk (M.B.); oj@nanonord.dk (O.N.J.)
[2] Department of Biological and Chemical Engineering, Aarhus University, Finlandsgade 12, DK-8200 Aarhus, Denmark
[3] Interdisciplinary Nanoscience Center (iNANO) and Department of Chemistry, Aarhus University, Gustav Wieds Vej 14, DK-8000 Aarhus, Denmark
* Correspondence: moks@bce.au.dk (M.K.S.); ncn@nanonord.dk (N.C.N.)
† Present address: Interdisciplinary Nanoscience Center (iNANO) and Department of Chemistry, Aarhus University, Gustav Wieds Vej 14, DK-8000 Aarhus, Denmark.

Table S1: List of supermarket milk products with declared contents, NMR results (mean of duplicate samples) and laboratory results for fat and protein, respectively. For protein, the declared contents are given as the package declaration scaled by $k = 6.38/6.25$, since package declarations are based on an assumed Jones factor of 6.25.

	Fat (%)			Protein (%)		
**Product description**	**Decl.**	**NMR**	**Lab**	**Decl.*k**	**NMR**	**Lab**
Stay strong protein drink, taste of vanilla. Homogenized, UHT-treated.	0.3	0.42		6.12	5.88	
Arla whole milk. Pasteurized and homogenized. June 2021.	3.5	3.60		3.47	3.48	
Arla organic low-fat milk. Pasteurized and homogenized.	0.4	0.40		3.57	3.26	
Ânglamark (Thise) organic whole milk. Pasteurized, not homogenized.	3.5	3.45		3.57	3.43	
Arla lactose-free whole milk. Pasteurized and homogenized.	3.5	3.58		3.37	3.30	
Thise organic jersey reduced-fat milk. From jersey cows. Pasteurized, not homogenized.	1.5	1.61		4.08	4.10	
Irma skimmed milk. Pasteurized and homogenized.	0.1	0.05	0.11	3.57	4.53	4.37
Thise organic 'grass milk'. From cows only fed with grass. Pasteurized, not homogenized.	4.2	4.07		3.78	3.69	
Coop coffee cream. Sterilized and homogenized.	9.0	9.04		3.16	3.39	
Thise 'primitive milk'. With high content of A2 protein, from jersey cows. Pasteurized, not homogenized.	0.5	0.62		4.19	4.37	
Douwe Egberts low-fat milk. Homogenized and UHT treated.	0.5	0.58		3.57	3.89	
Arla organic skimmed milk. Pasteurized, not homogenized.	0.1	0.09		3.57	3.73	
Arla whole milk. Pasteurized and homogenized. October 2021.	3.5	3.45	3.59	3.47	4.09	4.02
Mælkebøtte reduced-fat milk. Pasteurized and homogenized.	1.6	1.54		3.67	3.76	
Milsani long-life whole milk. Homogenized and UHT treated.	3.6	3.58		3.47	3.62	
Trak'n Eat Whole milk powder. Mixed 1:8 with H_2O as proposed by manufacturer.	2.9	2.99		3.06	2.78	
Andechser Natur long-life organic goat milk. Homogenized, UHT treated.	3.2	3.21		3.16	3.44	
Leep Vital long-life organic sheep milk. Homogenized, UHT treated.	6.0	4.99	5.12	4.70	4.59	4.45
Nestle NAN Pro 1, breast milk substitute.	3.6	3.42		1.22	1.61	
Semper Allomin 2, supplement mixture.	3.4	2.90	3.20	1.43	1.46	1.37
